# Dental implant treatment in a young woman after marginal mandibulectomy for treatment of mandibular gingival carcinoma: a case report

**DOI:** 10.1186/s40729-015-0022-2

**Published:** 2015-08-04

**Authors:** Kazuki Takaoka, Emi Segawa, Michiyo Yamamura, Yusuke Zushi, Masahiro Urade, Hiromitsu Kishimoto

**Affiliations:** Department of Oral and Maxillofacial Surgery, Hyogo College of Medicine, 1–1 Mukogawa-cho, Nishinomiya, Hyogo 663-8501 Japan

**Keywords:** Postoperative rehabilitation, Oral cancer, Implant-fixed prosthesis, Implant-supported overdenture

## Abstract

Dental implants play an important role in postoperative rehabilitation after surgical treatment of oral cancer through the provision of prosthetic tooth replacement. Two major implant prosthesis designs are available: fixed implant-supported prostheses and implant-supported overdentures. We herein report a case of a 16-year-old female patient who underwent alveolar ridge resection for treatment of mandibular gingival carcinoma. Following surgery, oral rehabilitation was attempted using an implant-supported overdenture on a gold bar retainer splinting four implants. However, the patient was not satisfied with this prosthesis because of mucosal pain and discomfort, and she gradually ceased its use. Consequently, contact with the opposing teeth caused wear of the prosthetic screws. We elected to replace the implant-supported overdenture with an implant-fixed prosthesis approximately 16 years after insertion of the overdenture to prevent further wear of the prosthetic screws. The patient was highly satisfied with the improved stability of the implant-fixed prosthesis. This case report indicates that the clinician must occasionally re-evaluate and sometimes alter the direction of treatment, even after definitive therapy has been completed.

## Background

Surgical treatment of oral cancer may lead to significant disability, including facial deformity, loss of hard and soft tissue, and impaired function of speech, swallowing, and mastication [1]. Bone resection because of surgical treatment of a large mandibular tumor can cause long-term defects. Rehabilitation with a removable prosthesis can be difficult or impossible due to the distorted postsurgical anatomy, especially for edentulous patients, for whom provision of a removable prosthesis is almost impossible. Dental implants are useful to improve the stability and support of a prosthesis, and dental implants have recently gained an important role in the rehabilitation of patients with oral cancer by facilitating the provision of a stable prosthesis [1]. Two major implant prosthesis designs are available: fixed implant-supported prostheses and implant-supported overdentures. Several factors affect the choice between fixed and removable implant prostheses, such as the interforaminal space, interjaw relationship, oral hygiene, cost, and patient preference [2]. Zani et al. [3] reported that both fixed implant-supported prostheses and implant-supported overdentures were perceived to be equally satisfactory by mandibular edentulous patients and that the condition of the prostheses did not influence individual satisfaction in terms of rehabilitation. In this clinical case, an implant-supported overdenture that was delivered to rehabilitate the edentulous mandibular region following marginal mandibulectomy for treatment of mandibular gingival carcinoma was replaced by an implant-fixed prosthesis. Different treatment pathways should be prepared during the treatment planning stage.

The purpose of this paper is to present a case report of dental implant placement in a 17-year-old female patient after marginal mandibulectomy for treatment of mandibular gingival carcinoma, subsequent prosthodontic treatment, and an almost 22-year follow-up after dental implant placement.

## Case presentation

**Fig. 1:**
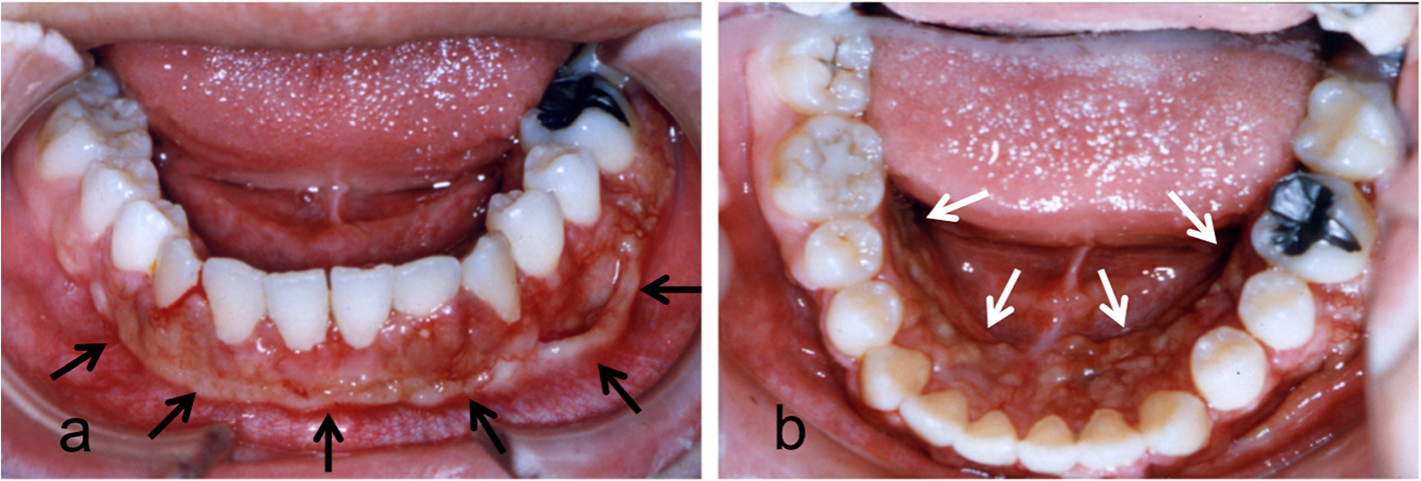
Intraoral photograph showing diffuse tumor formation on the alveolar gingiva (arrows)

**Fig. 2:**
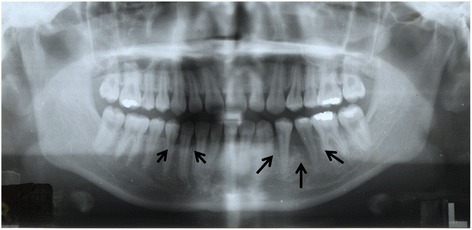
Panoramic radiograph showing notable alveolar bone resorption in the left mandibular premolar region and slight resorption in the right mandibular canine region (arrows)

**Fig. 3:**
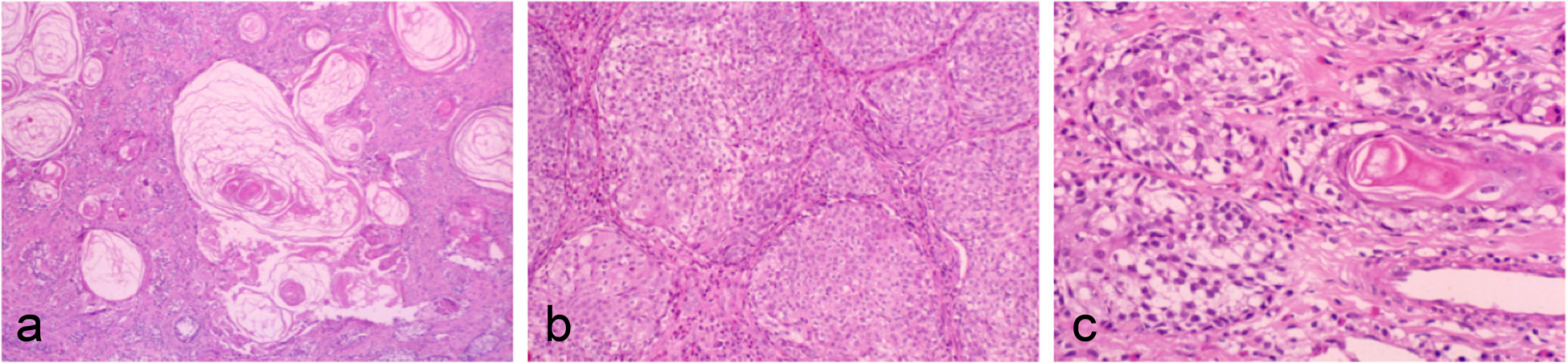
Photomicrographs of the biopsy specimen showing the intermingling of (**a**), (**b**), and (**c**). **a** Moderately differentiated epidermoid tumor cells with a duct-like structure (hematoxylin and eosin [H&E], original magnification × 100). **b** Intermediate cells (H&E, original magnification × 100). **c** Clear cells (H&E, original magnification × 100)

**Fig. 4:**
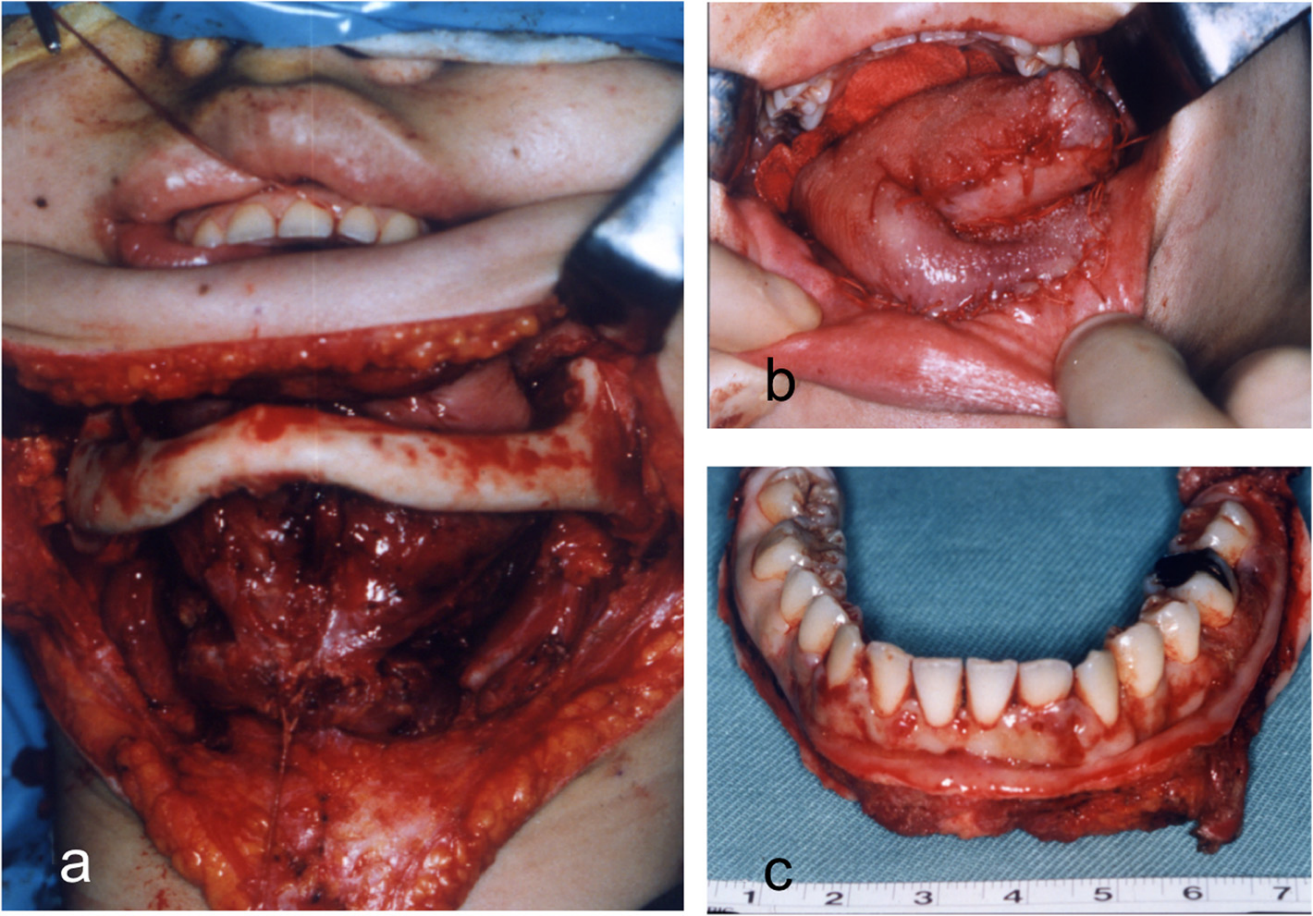
**a** Intraoperative photograph of resection of the alveolar ridge and bilateral upper neck dissection. **b** Transplantation of a lateral tongue flap to cover the alveolar ridge defect. **c** Surgical specimen

A 16-year-old female patient developed slight tenderness of the gingiva in the left mandibular premolar region, and her dentist referred her to our clinic in April 1992. Oral examination showed erythematous granular swellings that bled easily on the alveolar gingiva involving the area extending from the right second premolar to the left second molar (Fig. 1). The lesion showed extensive, superficial growth but was largely confined to the attached gingiva. Radiographic examination showed notable alveolar bone resorption in the left mandibular premolar region and slight resorption in the right mandibular canine region (Fig. 2). The lesion was biopsied, and histopathological examination showed diffuse unencapsulated proliferation of moderately to poorly differentiated squamoid tumor cells with occasional mitotic figures and keratinization as well as tumor nests comprising intermediate and clear cells. Few duct-like structures were observed, but microcysts were occasionally noted (Fig. 3). The specimen histologically resembled a mucoepidermoid carcinoma [4], but presented as a rare gingival carcinoma of the mandible. Treatment in May 1992 involved resection of the alveolar ridge between the right and left second molar regions, preserving the right inferior alveolar nerve, followed by bilateral upper neck dissection and transplantation of a lateral tongue flap to cover the alveolar ridge defect (Fig. 4). Histopathological examination of the surgical specimens showed bilateral submandibular lymph node metastases. Two courses of postoperative chemotherapy were performed to prevent local recurrence and distant metastasis.

**Fig. 5:**
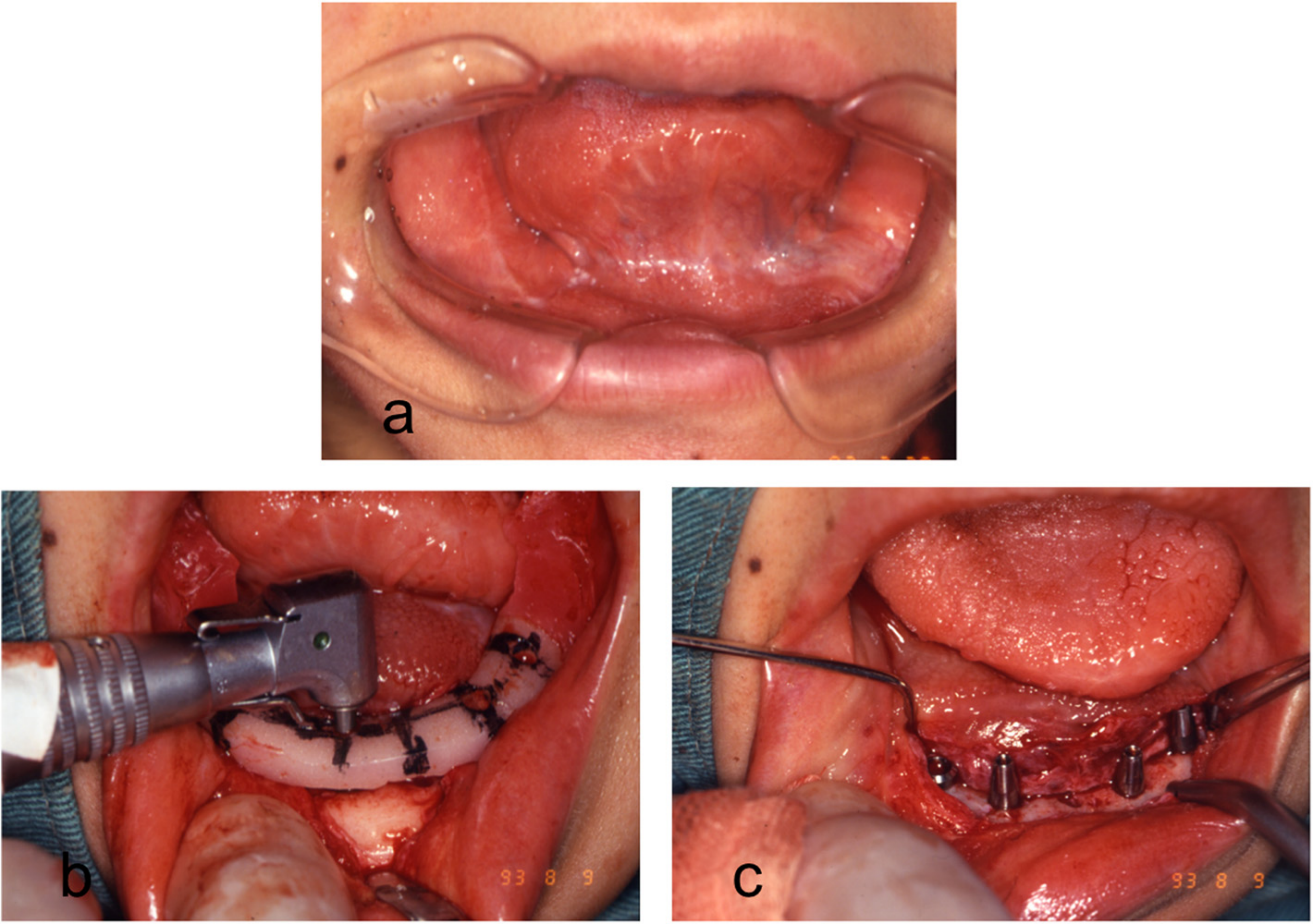
Preoperative intraoral photograph of implant placement

**Fig. 6:**
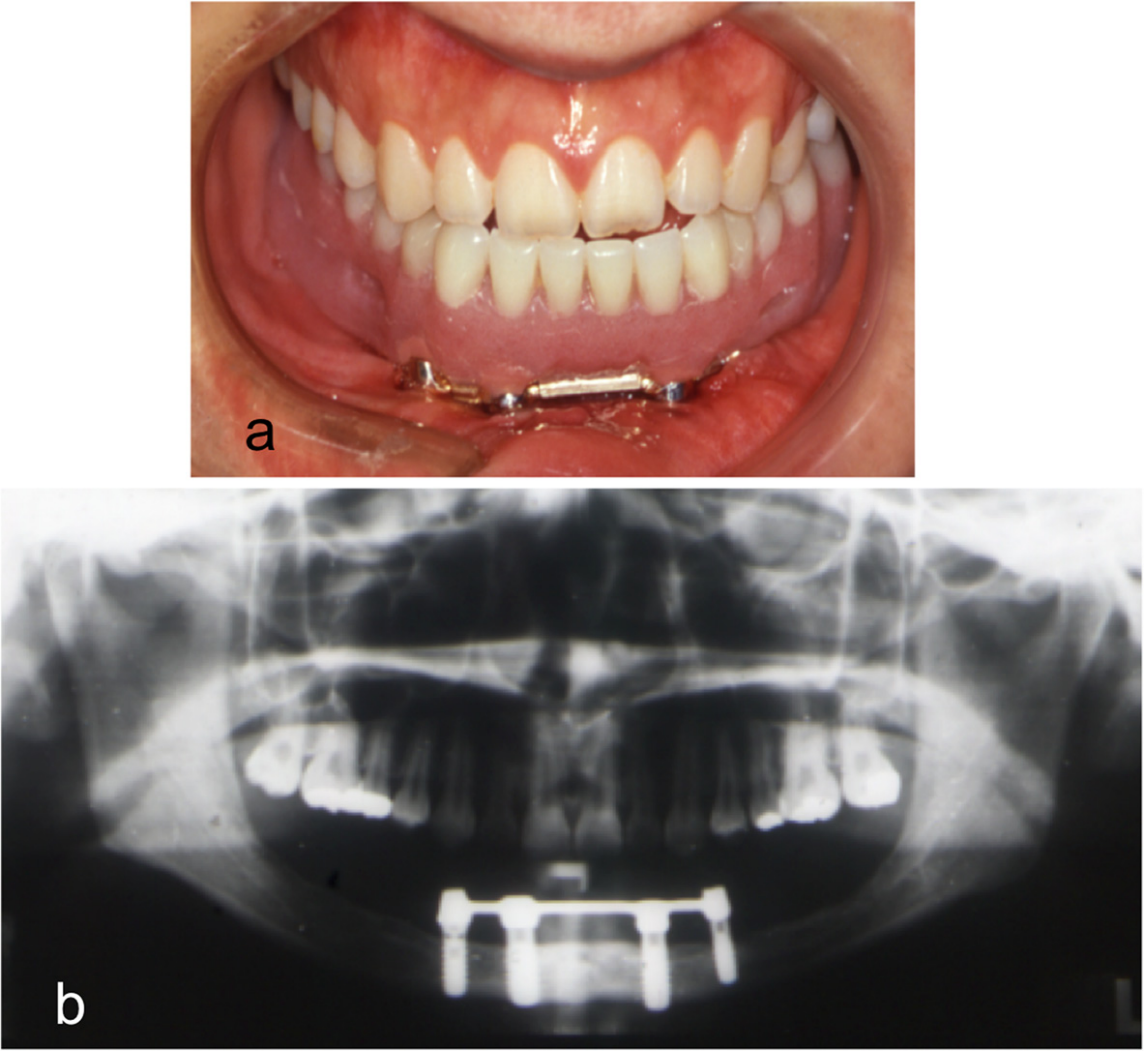
**a** Mandibular implant-supported overdenture inserted into the mouth. **b** Panoramic radiograph after insertion of the prosthesis

**Fig. 7:**
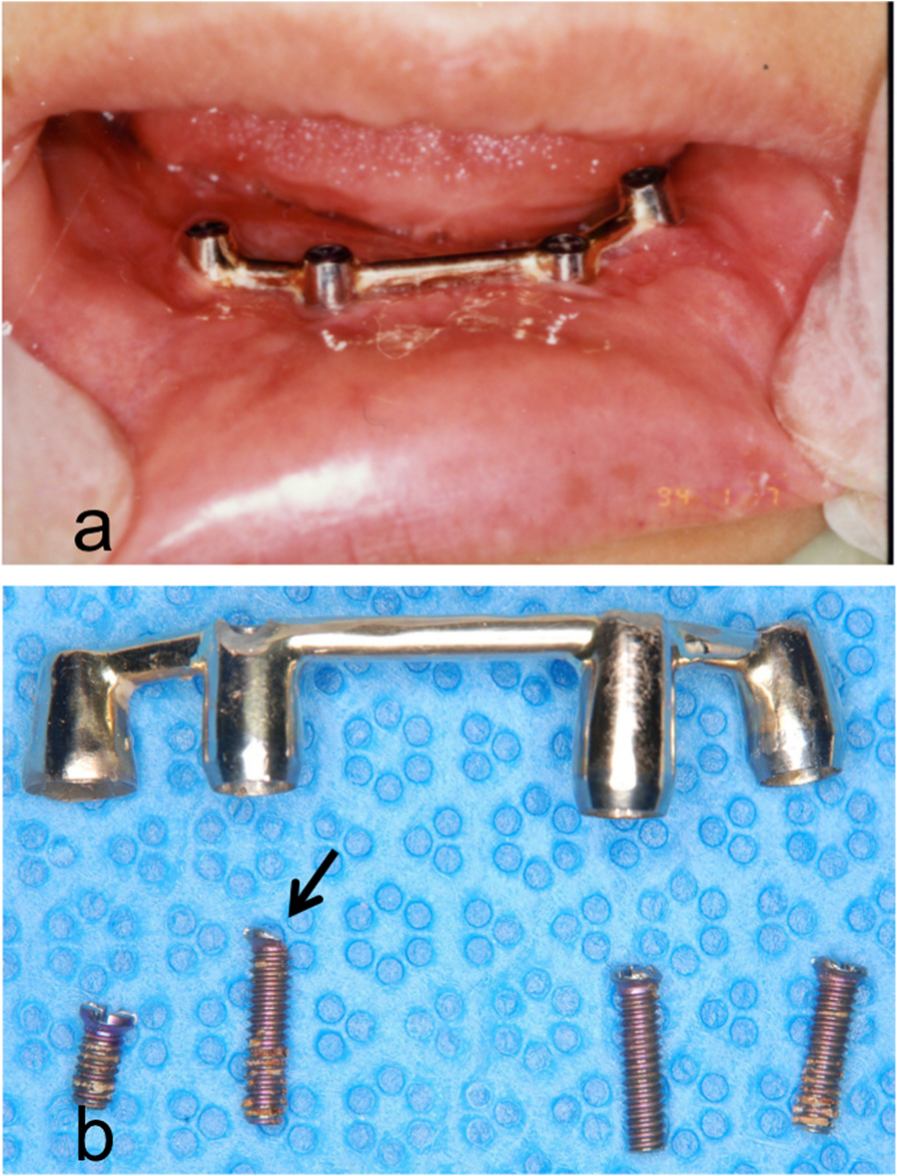
**a** Intraoral photograph. **b** Gold Dolder bar and screws; marked wear of a prosthetic screw (arrow)

**Fig. 8:**
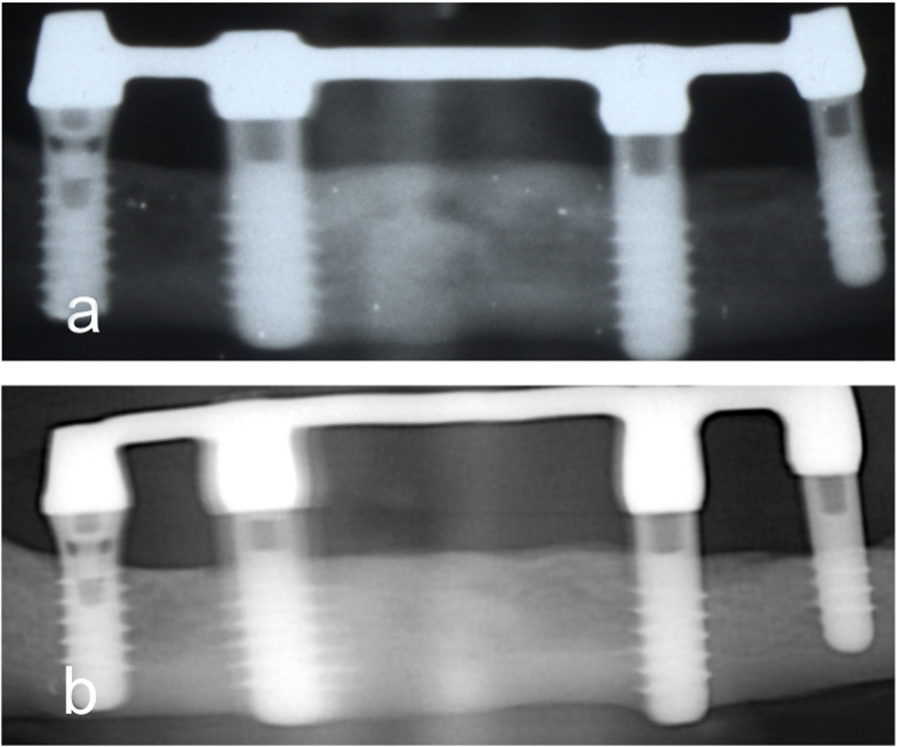
Periapical radiographs of the implants. **a** Postoperative, 1 year. **b** Postoperative, 16 years

There was no evidence of recurrence or metastasis at 1 year 3 months after surgery, and an International Team for Implantology (ITI) implant system (Institut Straumann AG, Basel, Switzerland) and prosthetic appliance were provided for cosmetic improvement and recovery of masticatory function. Four implants (three measuring 4.1 × 10 mm, one measuring 4.1 × 8 mm) were placed in the mandible in August 1993 (Fig. 5). Three months later, these implants were connected by three gold U-shaped Dolder bars soldered to gold copings and made to fit passively. The overdenture incorporated three corresponding riders/clips acting as matrices on the intaglio surface, providing attachments to the bar retainer. The implant-supported overdenture was inserted and adjusted until the patient felt no pain (Fig. 6). The patient was given oral hygiene instructions and scheduled for follow-up appointments. However, she was not satisfied with the prosthesis; she experienced denture discomfort and developed a decubital ulcer in the tongue flap area, and she gradually ceased use of the denture. The patient was followed for more than 10 years on a regular basis to examine recurrence or metastasis of the gingival carcinoma. Mild erythema and swelling of the mandibular and implant-surrounding mucosa secondary to stimulation during mastication were found (Fig. 7a), and contact with the opposing teeth resulted in wear of the prosthetic screws (Fig. 7b). In this case, the opposing occlusion involved the natural teeth, and the bone loss around the implants was negligible during the 15 years of follow-up (Fig. 8). We had previously proposed replacing the overdenture with an implant-fixed prosthesis; however, at that time, the patient elected not to proceed with this option because of the additional economic burden. Eventually, however, the patient opted for rehabilitation with a fixed implant-supported prosthesis. The patient was not willing to undergo extensive surgical intervention, including preprosthetic surgery and placement of additional implants. Therefore, we elected to replace the implant-supported overdenture with an implant-fixed prosthesis in May 2010.

**Fig. 9:**
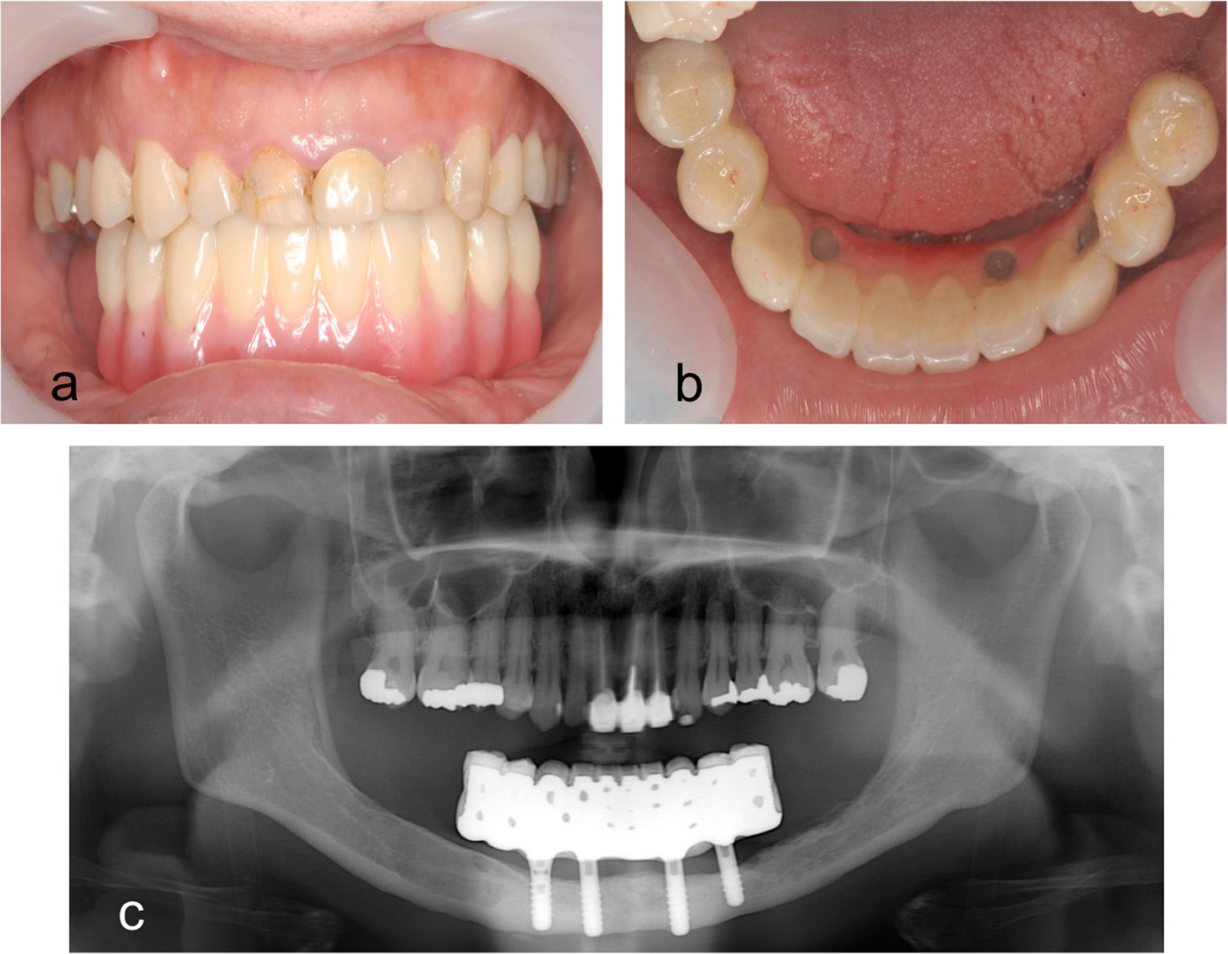
**a** Mandibular implant-fixed prosthesis inserted into the mouth. **b** Occlusal view of the implant-fixed prosthesis. **c** Panoramic radiograph 3 years 11 months after insertion of the fixed implant-supported prosthesis

After the impressions were taken, the implant-fixed prosthesis was fabricated with a cantilever design comprising two dental units. The mandibular prosthesis was then inserted in February 2011, and the final occlusion was verified and adjusted (Figs. 9a, b). The prosthesis was attached to the implants using prosthetic screws. The screw holes were filled with a dental temporary material overlaid with light-curable composite resin. The patient was highly satisfied with the improvement in oral rehabilitation as a result of the new prosthesis. She was instructed on brushing techniques and reviewed every 6 months. Neither dental plaque nor calculus beneath the prosthesis was detected, and there was no mucosal erythema or bone loss around the implants (Fig. 9c), which remained healthy almost 4 years after insertion of the final prosthesis.

## Conclusions

Prosthetic rehabilitation of edentulous patients after surgical management of oral cancer is difficult and therefore often avoided. However, adequate prosthetic rehabilitation is a pivotal factor for patients to regain oral function [5]. In terms of the masticatory rehabilitation of these patients, the application of a removable prosthesis unsupported by implants may be difficult or even impossible because of the postsurgical anatomical alteration [6]. The benefits of implant-supported prostheses have been recognized for several years [7]. Dental implants may improve denture retention and stability without unnecessary loading of the vulnerable mucosa. Function, comfort, esthetics, and eventually quality of life can be improved [8]. Two different options for oral rehabilitation using dental implants exist. One of these is the fixed prosthesis supported by implants, which does not involve any contact with the oral mucosa, thereby preventing frictional ulcers. The other option is an implant-supported overdenture, which allows improved oral hygiene [6]. Barão et al. [9] reported that patients with implant-supported overdentures exhibited a higher degree of stress on the supporting mucosa than those with fixed implant-supported prostheses. In those with fixed implant-supported prostheses, the prosthesis is completely supported by the implants, with no mucosal contact; therefore, fixed implant-supported prostheses limit the degree of mechanical irritation to the soft tissue.

Based on the clinical and histological findings, our case was considered to be an intermediate-grade mucoepidermoid carcinoma. Because wide local surgical excision is critical in the treatment of this tumor, we performed entire resection of the alveolar ridge, also considering her age and esthetic concerns. Loss of the alveolar ridge led to severe masticatory dysfunction. In the present case, the patient refused further surgical intervention following surgical removal of the gingival carcinoma, and we adopted an implant-supported overdenture because of its relative simplicity, ease of self-maintenance, and affordability. According to the literature, in patients with malignancies involving the lower region of the oral cavity, a minimum of four implants is needed to achieve maximal implant support for the prosthesis and to relieve the vulnerable underlying soft tissues [10, 11]. We inserted an implant-supported overdenture on a gold bar retainer splinting four implants. However, the patient was not satisfied with this prosthesis because of the mucosal pain and discomfort that developed over time. In such cases, prosthetic loading of atrophic mucosa is often not well tolerated. As such, we proposed replacement with an implant-fixed prosthesis. Initially, the patient elected not to proceed with this option because of the additional economic burden. However, the patient eventually opted for rehabilitation with a fixed implant-supported prosthesis, as this provided the psychological advantage of a prosthesis that felt similar to the natural teeth. In this case, an implant-supported overdenture, which was provided to rehabilitate the edentulous mandibular region after marginal mandibulectomy for treatment of gingival carcinoma of the mandible, was replaced by an implant-fixed prosthesis.

Pjetursson et al. [12] performed a systematic review of the survival and complication rates of implant-fixed prostheses after a mean observation period of at least 5 years. They concluded that implant-fixed prostheses are a safe and predictable treatment method with high survival rates. However, biological and technical complications were frequent in their review (33.6 %). To minimize the incidence of complications, dental professionals should make great effort to choose reliable components and materials for implant-fixed prostheses, and patients should undergo a well-structured maintenance protocol after treatment. In the present case, professional teeth cleaning with individual instruction every 3 months improved the patient’s oral hygiene. Maintenance care may have motivated the patient to improve her oral home care regimen. This case report indicates that occasionally, even after definitive therapy has been completed, the clinician must re-evaluate and sometimes alter the direction of treatment to provide the best possible outcome for the patient. In conclusion, we have herein reported a case illustrating our long-term clinical experience and the concept of switching therapy.

## Consent

Written informed consent was obtained from the patient for publication of this case report and any accompanying images. A copy of the written consent is available for review by the Editor-in-Chief of this journal.
